# Construction and evaluation of a risk prediction model for work-related musculoskeletal disorders among construction workers at a hydropower station on the Qinghai-Tibet Plateau: a cross-sectional study

**DOI:** 10.3389/fpubh.2026.1850053

**Published:** 2026-07-02

**Authors:** Anran Jiang, Yaoqi Li, Deji Zhaxi, Duxuan Zhong, Yanbiao Bai, Xingzhang Luo, Ci Song

**Affiliations:** Medical College, Xizang University, Lhasa, China

**Keywords:** construction workers, LASSO regression, nomogram, Qinghai-Tibet Plateau, work-related musculoskeletal disorders (WRMSDs)

## Abstract

**Introduction:**

The use of outdated building models and inefficient management practices continue to pose serious risks to the occupational health of workers in China’s rapidly expanding construction sector. We identified key risk factors for work-related musculoskeletal disorders (WRMSDs) among hydropower station construction workers in the Qinghai-Tibet Plateau and developed a risk prediction model.

**Methods:**

This cross-sectional survey of 325 workers was conducted in December 2025. Least absolute shrinkage and selection operator regression was used for variable selection and multivariate logistic regression was employed to construct a prediction model visualized as a nomogram. Model performance was validated using receiver operating characteristic, calibration, and decision curve analysis.

**Results:**

The prevalence of neck, shoulder, and lower back WRMSDs was 31.1% (101/325). Five key predictors were identified: household registration type, chronic disease history, sleep duration, work type, and workplace temperature. The model demonstrated good discrimination (area under the curve = 0.789, 95% confidence interval 0.724–0.853) and calibration (*p* > 0.05). Urban household registration (odds ratio *OR* = 2.24, *p* = 0.036), electromechanical installation work (*OR* = 3.62, *p* = 0.011), other occupations (*OR* = 5.56, *p* < 0.001), chronic disease history (*OR* = 6.02, *p* = 0.018), and cold workplace (*OR* = 2.47, *p* = 0.006) were risk factors, whereas longer sleep duration was a protective factor (*OR* = 0.80, *p* = 0.049).

**Discussion:**

In this population, WRMSDs were influenced by demographic, health, and environmental factors. The developed model enables early identification of high-risk workers and supports targeted occupational health interventions.

## 1 Introduction

Despite the rapid development of China’s construction industry, extensive construction models and lagging management practices continue to pose significant threats to workers’ occupational health (1). Work-related musculoskeletal disorders (WRMSDs) are diseases characterized by injury or dysfunction of muscles, bones, nerves, joints, cartilage, tendons, and ligaments caused by exposure to harmful factors during occupational activities. Symptoms include numbness, burning sensations, tingling, and limited mobility, which pose a serious threat to the health of the working population (2). WRMSDs are characterized by high incidence rates and broad industrial coverage, making them a major global occupational health concern. In most industrialized countries, their incidence ranks second to occupational mental illnesses, making them the second most common occupational disorder (3, 4). The pathogenesis of WRMSDs is complex and encompasses three dimensions: individual physiological, psychosocial, and occupational ergonomic factors (5). WRMSDs are prevalent occupational health issues in the construction industry, with particularly pronounced hazards in plateau environments (6). Unique plateau conditions such as hypoxia, low temperatures, and intense radiation, combined with high-intensity physical labor, exacerbate the burden on the musculoskeletal system and may alter the mechanisms and clinical manifestations of WRMSDs (7). Hypoxic environments can reduce the body’s metabolism and recovery capacity, accelerating muscle fatigue. Further, low temperatures can impede local blood circulation, increasing the risk of joint and soft tissue injuries (8, 9).

The prevalence of WRMSDs among construction workers varies significantly across anatomical regions, with the lumbar region, shoulders, neck, and knees being high-incidence and most vulnerable sites for musculoskeletal injuries in construction workers (10, 11). Additionally, construction workers at high-altitude hydropower stations are commonly subjected to high-intensity physical labor and prolonged occupational vibration exposure, conditions which are well-established triggers of musculoskeletal injury. Studies on mining populations have shown that long-term whole-body vibration exposure significantly increases the risk of lumbar injury and chronic lower back pain. Biomechanical models further confirm that vibration can lead to spinal overload and tissue degeneration (12, 13). In high-altitude construction settings, hypoxia, low temperatures, and vibration exposure may produce a synergistic effect and exacerbate musculoskeletal damage; however, such risk mechanisms remain unclear.

On the eastern edge of the Qinghai-Tibet Plateau, the special natural environment of hypoxia, low temperatures, and high radiation intertwines with occupational hazard factors associated with hydropower station construction, such as high-intensity physical labor, dust, noise, and toxic substances (14). This poses severe challenges to the physical and mental health of long-term on-site construction personnel, especially those working on lowland areas. However, existing research has primarily focused on plains or conventional working environments, and systematic investigations of WRMSDs among construction workers in plateau environments with unique conditions remain relatively scarce. Considering that WRMSDs predominantly cluster in the lumbar, cervical, and shoulder regions, establishing a targeted risk prediction model for these high-incidence sites is meaningful for the prevention and control of occupational hazards (15). Therefore, in this study, we aimed to conduct an epidemiological survey among construction workers at a hydropower station on the Qinghai-Tibet Plateau, systematically identify key risk factors for WRMSDs, assess their health impacts, and construct a risk prediction model.

## 2 Materials and methods

### 2.1 Participants

In December 2025, we included 331 workers at a hydropower station for this study. We included (1) participants who provided informed consent and (2) those aged >18 years. We excluded (1) participants who were unable to understand the questionnaire content or answer some survey questions; (2) those with a history of severe organic diseases or musculoskeletal disorders caused by tumors, trauma, infection and other non-occupational reasons; (3) those with a history of mental illness, recent history of taking psychotropic drugs, or family history of mental illness; and (4) those with sleep disorders caused by other diseases such as post-traumatic stress disorder. Of 331 questionnaires distributed, 325 valid responses were obtained, with an effective response rate of 98.2%. This study was approved by the Ethics Committee of the Medical College of Xizang University (opinion number: ZDYXLL2025025), and all participants signed an informed consent form.

### 2.2 Methods

#### 2.2.1 Definition of WRMSDs

The survey was conducted using the Chinese Musculoskeletal Questionnaire (CMQ), which was revised by Yidan et al. (16) based on the actual situation in China. CMQ consists of 48 items across nine dimensions, with composite reliability and convergent validity ranging from 0.54 to 0.93 and 0.377 to 0.834, respectively; this indicates good reliability and validity. The survey content was used to assess participants’ experiences of pain or discomfort in nine body regions (neck, shoulders, back, elbows, wrists, lower back, hips/buttocks, knees, and ankles/feet) over the past year, as well as work-related factors such as heavy load lifting, repetitive movements, and rest adequacy. Ultimately, the outcome variable was defined as the presence of discomfort, numbness, or pain in the lower back, neck, or shoulder regions since the start of work, with symptoms lasting >24 h, failing to resolve with rest, and confirmed by a professional physician’s diagnosis. Cases caused by tumors, tuberculosis, or trauma we excluded.

#### 2.2.2 Baseline demographic characteristics

We adopted a cross-sectional epidemiological survey method for this study. A questionnaire designed by the research group, based on literature review, expert consultation, and content discussion, was used as the survey tool to collect information. We collected information on (1) general social demographic characteristics (sex, age, ethnicity, household registration, education level, income status, marital status, history of chronic diseases); (2) behavioral characteristics (physical exercise, smoking, drinking, sleep duration); (3) work organization characteristics (occupation type, years of work experience, high-altitude working time, daily working hours, shift work, labor intensity, workplace temperature, environmental noise); and (4) survey of injury symptoms in the past year to judge the severity of WRMSDs. History of chronic diseases was based on workers’ reported disease histories such as hypertension, diabetes and chronic obstructive pulmonary disease diagnosed by professional doctors.

#### 2.2.3 Occupational-related definitions

Given the unique characteristics of the construction environment of high-altitude hydropower stations, on-site occupations were classified into six categories: civil construction (e.g., concreters, steel fixers, scaffolders), electromechanical installation (e.g., electricians, welders, fitters), machinery operation (e.g., excavator drivers, loaders, rivers, road roller drive operators), basic support work (general laborers, surveyors, signal men), administrative management, and others (drivers of small vehicles, security posts, kitchen and other service positions). Workplace temperature was divided into comfortable, cold, and hot; noise was divided into work-impairing and non-work-impairing. Regarding work schedules, data on weekly working hours were collected via the questionnaire.

#### 2.2.4 Physical examination

All surveyors received standardized training and followed standardized operating procedures. Core measurements included height, body weight, waist circumference, blood pressure, oxygen saturation, heart rate, electrocardiogram (ECG), and lumbar spine bone mineral density (BMD). Height and body weight were measured using calibrated stadiometers and scales, with precisions of 0.1 cm and 0.1 kg, respectively. Blood pressure was measured using a calibrated Omron upper-arm electronic sphygmomanometer (HEM-1000). Participants were required to sit and rest for 5–10 min before measurement. Blood pressure was measured on the right upper arm in a seated position and the average of two consecutive measurements was taken. After participants had rested in seated positions for 5 min, oxygen saturation and heart rate were measured using a finger pulse oximeter. Standard 12-lead ECG results were obtained at rest. Lumbar spine BMD was assessed using dual-energy X-ray absorptiometry. All measurement instruments were uniformly calibrated to ensure data accuracy and reliability. Body mass index (BMI) was calculated as BMI = body weight (kg) divided by height^2^ (m^2^). BMD results were classified into normal bone mass, osteopenia, and osteoporosis.

### 2.3 Quality control

Investigators were uniformly trained before the survey and on-site organization was performed by cooperating parties. After the investigators explained the requirements, participants were guided to fill the online questionnaire. After data collection, the questionnaires were immediately checked, assigned unique identification numbers, and subjected to logical consistency checks. Invalid questionnaires were excluded to ensure the authenticity and reliability of the data.

### 2.4 Statistical analysis

Data were analyzed using SPSS 27.0 and R 4.5.2 software. Missing data were addressed using multiple imputations. Measurement data with non-normal distribution were expressed as M (P25, P75), and the Kruskal–Wallis *H* test was used for inter-group comparison; categorical variables are expressed as frequency and percentage (n, %), and the chi-square or Fisher’s exact probability tests were used for between-group comparisons. The Kruskal–Wallis *H* test was used for ordinal data. The study sample was randomly divided into training (*n* = 227) and validation (*n* = 98) sets at a ratio of 7:3 for model development and internal validation. The model was constructed and fitted in the training set and its performance was evaluated in the validation set. All variables were included in the least absolute shrinkage and selection operator (LASSO) regression model, which was performed using the generalized linear models via elastic net package in R version 4.5.2. L1 regularization coupled with 10-fold cross-validation was used for variable screening. Variables with non-zero coefficients were selected as predictors by considering *λ*_min_ (corresponding to the minimum cross-validation error) and *λ*_1se_ (the simplest model within 1 standard deviation range, in which variables with non-zero coefficients were screened and retained as predictors). The screened variables were included in multivariate logistic regression analysis to construct a prediction model. Based on the training set, a nomogram was plotted using the regression modeling strategies package in RStudio. Model discrimination was evaluated using the area under the receiver operating characteristic (ROC) curve (AUC) in the training and validation sets. Model calibration was assessed using calibration curves with 1,000 bootstrap resamples for smoothing and the Hosmer-Lemeshow test (*p* > 0.05, indicated good goodness of fit). Decision curve analysis (DCA) was applied to evaluate the net clinical benefit of the prediction model. All statistical tests were two-sided, with a test level of *α* = 0.05.

### 2.5 Ethics statement

This study was approved by the Medical Ethics Review Committee of XiZang University (opinion number: ZDYXLL2025025). This study was conducted in accordance with the principles of the Declaration of Helsinki and requirements of local legislation and institutional guidelines. All participants provided informed consent prior to enrollment.

## 3 Results

### 3.1 Prevalence of WRSDs and univariate analysis

Overall, 325 construction workers at a hydropower station were included in this study; 101 participants showed neck, shoulder, and waist WRMSD symptoms, with an overall detection rate of 31.1%. The results of the univariate analysis showed significant differences in the detection rate of WRMSDs among participants with different sexes, household registration types, education levels, occupation types, history of chronic diseases, sleep duration, weekly working hours, workplace temperature, and noise exposure (*p* < 0.05). The prevalence rate in females (52.0%) was significantly higher than that in males (29.3%); the prevalence rate was highest among those with college education or higher (49.4%). In terms of work-related factors, the detection rate among administrative management personnel was as high as 53.8%, which was significantly higher than that among civil construction workers (10.5%) (Table 1). There were no significant differences in sex, age, ethnicity, registered residence type, education level, marital status, personal monthly income, or other related variables between the training and validation sets (*p* > 0.05) (Supplementary Table 1).

**Table 1:** Prevalence of WRMSDs and univariate analysis.

Variables	Number of participants	Group without neck, shoulder, or waist symptoms	Group with neck, shoulder, or waist symptoms	Chi-square (*χ*^2^)/*Z*	*p*-value
Sex				*χ*^2^ = 5.535	0.019
Male	300 (92.3)	212 (94.6)	88 (87.1)		
Female	25 (7.7)	12 (5.4)	13 (12.9)		
Age	41 (33, 49)	40 (33, 49)	41 (33, 50)	*Z* = −0.330	0.741
Ethnicity				*χ*^2^ = 1.994	0.369
Han	284 (87.4)	192 (85.7)	92 (91.1)		
Yi	21 (6.5)	17 (7.6)	4 (4.0)		
Other	20 (6.2)	15 (6.7)	5 (5.0)		
Registration place type				*χ*^2^ = 19.266	<0.001
Rural	272 (83.7)	201 (89.7)	71 (70.3)		
Urban	53 (16.3)	23 (10.3)	30 (29.7)		
Education Level				*χ*^2^ = 23.411	<0.001
Primary school and lower	59 (18.2)	46 (20.5)	13 (12.9)		
Junior high school	131 (40.3)	104 (46.4)	27 (26.7)		
High school/Secondary school	50 (15.4)	31 (13.8)	19 (18.8)		
Junior college and higher	85 (26.2)	43 (19.2)	42 (41.6)		
Marital Status				*χ*^2^ = 3.577	0.167
Unmarried	65 (20.0)	46 (20.5)	19 (18.8)		
Married	240 (73.8)	168 (75.0)	72 (71.3)		
Other	20 (6.2)	10 (4.5)	10 (9.9)		
Personal Monthly Income				*χ*^2^ = 3.699	0.157
≤7,000	86 (26.5)	64 (28.6)	22 (21.8)		
7,001–10,000	154 (47.4)	108 (48.2)	46 (45.5)		
≥10,001	85 (26.2)	52 (23.2)	33 (32.7)		
Physical Exercise				*χ*^2^ = 8.494	0.075
Never	141 (43.4)	89 (39.7)	52 (51.5)		
1–3 times per quarter	49 (15.1)	35 (15.6)	14 (13.9)		
2–3 times per month	48 (14.8)	39 (17.4)	9 (8.9)		
1–2 times per week	52 (16.0)	33 (14.7)	19 (18.8)		
≥3 times per week	35 (10.8)	28 (12.5)	7 (6.9)		
Smoking Status				*χ*^2^ = 0.509	0.775
Non-smoker	142 (43.7)	98 (43.8)	44 (43.6)		
Smoker	166 (51.1)	113 (50.4)	53 (52.5)		
Quitted smoking	17 (5.2)	13 (5.8)	4 (4.0)		
Drinking status				*χ*^2^ = 4.642	0.200
Never drink	128 (39.4)	97 (43.3)	31 (30.7)		
≤1 time per month	85 (26.2)	55 (24.6)	30 (29.7)		
2–4 times per month	81 (24.9)	52 (23.2)	29 (28.7)		
≥2 times per week	31 (9.5)	20 (8.9)	11 (10.9)		
Type of work				*χ*^2^ = 37.643	<0.001
Civil	86 (26.5)	77 (34.4)	9 (8.9)		
Electromechanical	51 (15.7)	33 (14.7)	18 (17.8)		
Machine Ops	20 (6.2)	16 (7.1)	4 (4.0)		
General Support	65 (20.0)	47 (21.0)	18 (17.8)		
Admin	52 (16.0)	24 (10.7)	28 (27.7)		
Other	51 (15.7)	27 (12.1)	24 (23.8)		
High-altitude work duration (months)	6 (3, 12)	6 (2, 12)	7 (4, 13)	*Z* = −1.446	0.148
History of chronic diseases				*χ*^2^ = 3.640	<0.001
None	312 (96.0)	221 (98.7)	91 (90.1)		
Yes	13 (4.0)	3 (1.3)	10 (9.9)		
Sleep duration (h)	7.5 (7, 8)	8 (7, 8)	7 (6, 8)	*Z* = −4.611	<0.001
Weekly working hours (h)	63 (56, 84)	63 (56, 77)	70 (56, 84)	*Z* = −3.489	<0.001
Workplace temperature				*χ*^2^ = 17.362	<0.001
Comfortable	161 (49.5)	120 (53.6)	41 (40.6)		
Hot	78 (24.0)	60 (26.8)	18 (17.8)		
Cold	86 (26.5)	44 (19.6)	42 (41.6)		
Work environment noise				*χ*^2^ = 4.128	0.042
No impact	285 (87.7)	202 (90.2)	83 (82.2)		
Impairs work	40 (12.3)	22 (9.8)	18 (17.8)		
Average heart rate	82 (73, 90)	81 (72, 89)	84 (75, 91)	*Z* = −1.261	0.207
Average blood oxygen	92 (91, 94)	92 (91, 94)	92 (91, 94)	*Z* = −0.227	0.820
Average systolic blood pressure	130 (120, 142)	130 (119, 143)	128 (122, 141)	*Z* = −0.303	0.762
Average diastolic blood pressure	84 (76, 94)	83 (76, 95)	86 (78, 92)	*Z* = −0.687	0.492
Bone mineral density (BMD)				*χ*^2^ = 1.614	0.446
Normal bone mass	165 (50.8)	110 (49.1)	55 (54.5)		
Osteoporosis	20 (6.2)	16 (7.1)	4 (4.0)		
Osteopenia	140 (43.1)	98 (43.8)	42 (41.6)		
Binary classification of ECG				*χ*^2^ = 0.025	0.873
Normal ECG	240 (73.8)	166 (74.1)	74 (73.3)		
Abnormal ECG	85 (26.2)	58 (25.9)	27 (26.7)		

### 3.2 Variable selection: LASSO regression analysis

**Figure 1:**
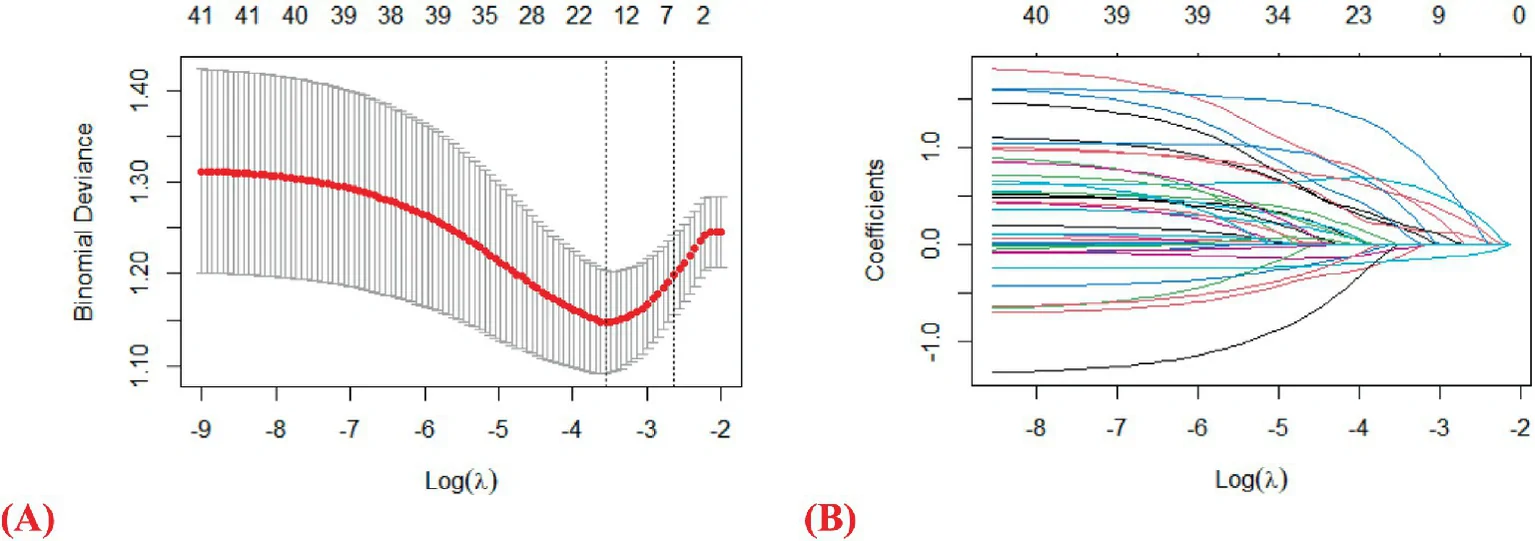
Screening the potential predictors through LASSO regression model. **(A)** Optimal lambda selection in the LASSO model. Dotted vertical lines were drawn at the optimal values. **(B)** LASSO coefficient profiles of the 24 features. LASSO, least absolute shrinkage and selection operator.

In the LASSO regression model, six key predictive variables, including household registration type, education level, history of chronic diseases, sleep duration, weekly working hours, and workplace temperature were selected (Figure 1). These variables were included in the multivariate logistic regression analysis. However, consistent with domestic and international research evidence, occupation type is a well-established risk factor for WRMSDs, with clear occupational exposure mechanisms and clinical significance. Therefore, it was additionally incorporated into the model (17, 18).

### 3.3 Multivariate logistic regression analysis

The risk of neck, shoulder, and waist WRMSDs among workers with urban household registration was 2.24 times that of those with rural household registration (odds ratio [OR] = 2.24, 95% confidence interval [CI]: 1.05–4.76, *p* = 0.036). Workers engaged in electromechanical installation (*OR* = 3.62, 95% CI: 1.35–9.68; *p* = 0.011) and other occupations (*OR* = 5.56, 95% CI: 2.12–14.59, *p* < 0.001) had significantly higher risks. Workers with a history of chronic diseases had a 6.02 times higher risk (*OR* = 6.02, 95% CI: 1.35–26.78, *p* = 0.018). Compared with workers in a comfortable environment, those who subjectively felt the workplace was “cold” had an increased risk (*OR* = 2.47, 95% CI: 1.30–4.69, *p* = 0.006). Sufficient sleep duration was a protective factor for WRMSDs (*OR* = 0.80, 95% CI: 0.64–0.99, *p* = 0.049) (Table 2).

**Table 2:** LASSO-logistic regression analysis for multiple factors.

Independent variable	Option	*β*	SE	Wald *χ*^2^	OR (95% CI)	*P*
Household registration type	Rural				1	
	Urban	0.806	0.384	4.397	2.24 (1.05–4.76)	0.036
Education level	Primary or lower				1	
	Junior high	−0.105	0.428	0.060	0.90 (0.39–2.08)	0.806
	High school	0.071	0.497	0.020	1.07 (0.41–2.84)	0.887
	Junior college	0.169	0.563	0.090	1.18 (0.39–3.57)	0.764
Type of work	Civil				1	
	Electromechanical	1.285	0.502	6.547	3.62 (1.35–9.68)	0.011
	Machine Ops	0.461	0.692	0.444	1.59 (0.41–6.15)	0.505
	General Support	0.631	0.501	1.585	1.88 (0.70–5.02)	0.208
	Admin	1.173	0.633	3.435	3.23 (0.94–11.18)	0.064
	Other	1.715	0.492	12.123	5.56 (2.12–14.59)	<0.001
History of chronic diseases	None				1	
	Yes	1.795	0.762	5.555	6.02 (1.35–26.78)	0.018
Workplace temperature	Comfortable				1	
	Hot	0.256	0.377	0.460	1.29 (0.62–2.70)	0.498
	Cold	0.903	0.328	7.569	2.47 (1.30–4.69)	0.006
Sleep duration		−0.226	0.115	3.869	0.80 (0.64–0.99)	0.049
Weekly working hours		0.007	0.008	0.673	1.01 (0.99–1.02)	0.412

### 3.4 Construction and visualization of risk prediction model

**Figure 2:**
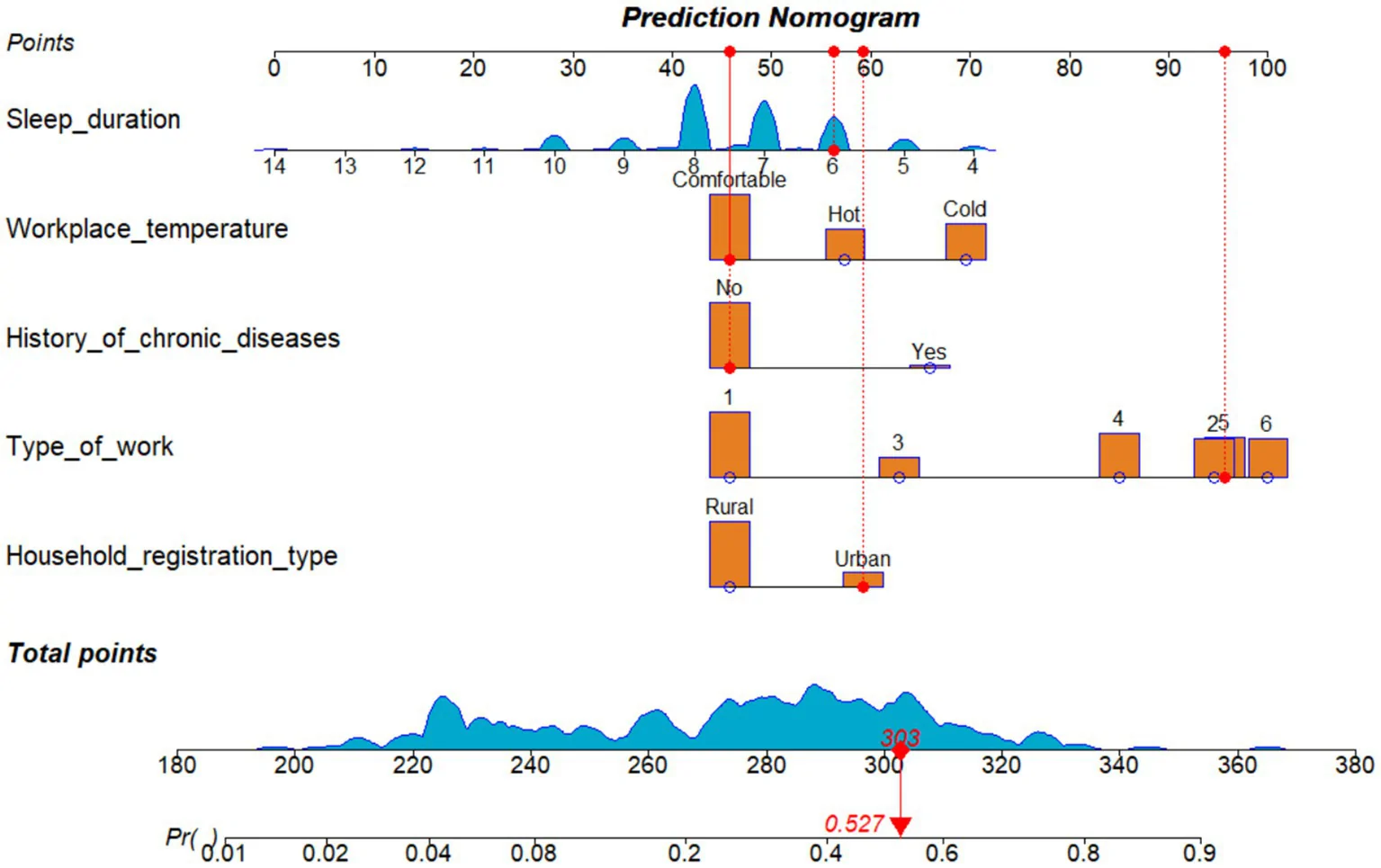
Nomogram for predicting the risk of WRMSDs among construction workers. Type of work: 1 = Civil work, 2 = Electromechanical installation, 3 = Machine operation, 4 = General support, 5 = Administration, 6 = Others. WRMSDs, work-related musculoskeletal disorders.

Based on the LASSO logistic regression results, a nomogram incorporating five variables was developed to predict risk (Figure 2). The final model included 101 outcome events, yielding an events per variable (EPV) ratio of 20.2, which exceeded the minimum standard of 10 EPV required for reliable logistic regression models. The final logistic regression model for predicting the risk of lumbocervicoscapular WMSDs among plateau hydropower employees is specified below:

Logit (*P*) = −0.968 + 0.806 × household registration type + 1.285 × electromechanical + 1.715 × other occupations + 1.795 × History of chronic diseases + 0.903 × workplace temperature − 0.226 × sleep duration.

This nomogram intuitively quantifies the contribution of each risk factor to disease risk and enables rapid individual risk assessment in field settings.

### 3.5 Validation and evaluation of the prediction model

**Figure 3:**
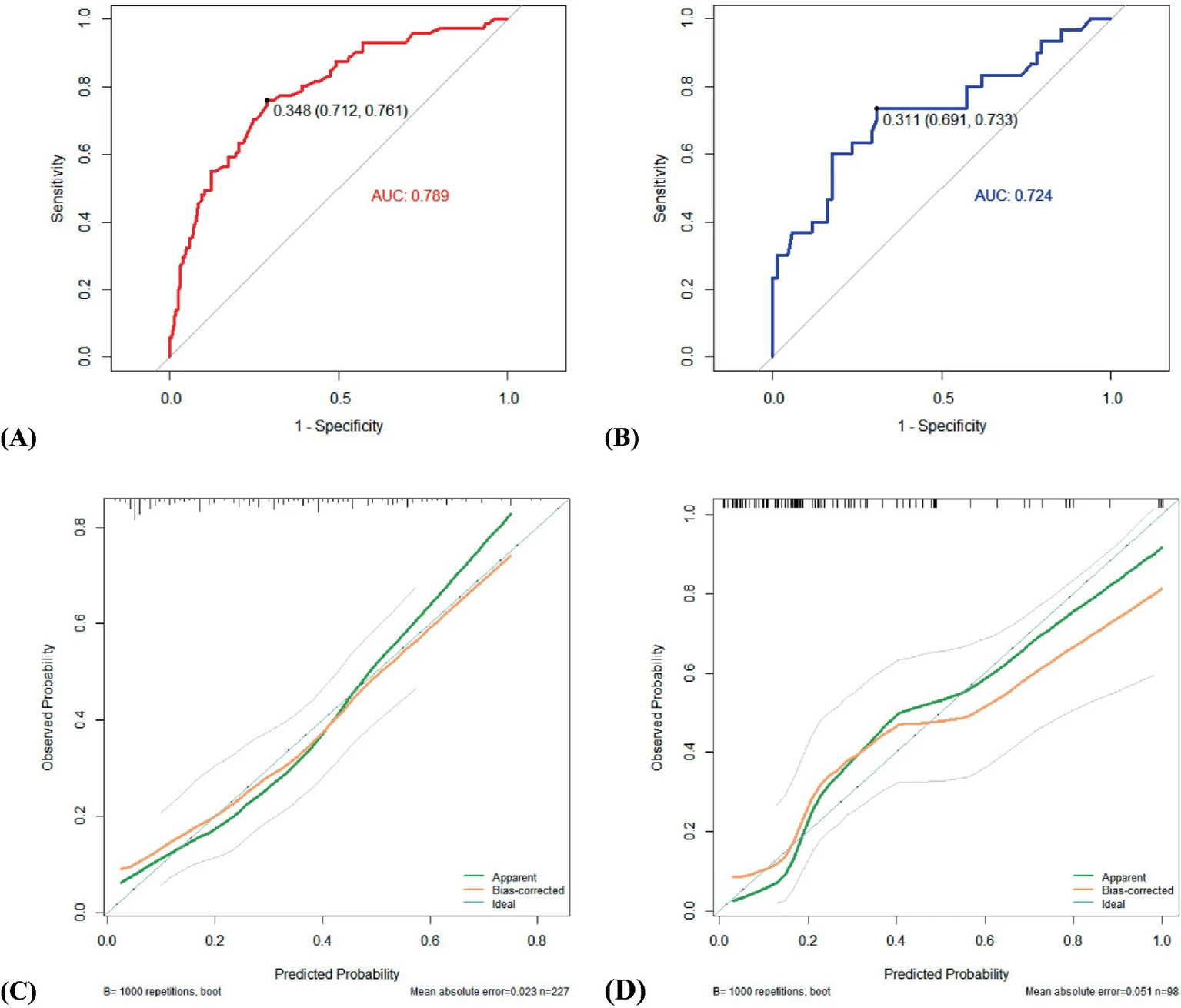
ROC and calibration curves of the training and validation sets. **(A,B)** The nomogram yielded an AUC of 0.789 (95% CI: 0.724–0.853) in the training set **(A)** and 0.724 (95% CI: 0.605–0.843) in the validation set **(B)**. **(C,D)** Calibration curves: the *x*- and *y*-axes indicate the predicted WRMSD risks and actual WRMSD prevalences, respectively. The diagonal dashed line represents perfect prediction by an ideal model; the solid line represents the nomogram performance, with a closer fit to the dashed line indicating better predictive accuracy. AUC; area under the curve; CI, confidence interval; ROC, receiver operating characteristic; WRMSD, work-related musculoskeletal disorder.

The ROC curve was used to evaluate the discriminative ability of the model, and the results showed that the AUC of the model was 0.789 (95% CI: 0.724–0.853), indicating that the model had good discriminative ability (Figure 3A). The calibration curve showed good consistency between the predicted and actual values, and the results of the Hosmer-Lemeshow test indicated an adequate goodness of fit of the model (*p* > 0.05), confirming the reliable calibration performance of the model (Figure 3). Data points (0.0, 0.0), (0.2, 0.2), (0.4, 0.4), (0.6, 0.6), and (0.8, 0.8) were distributed closely along the diagonal line, with the solid line fitting well with the dotted diagonal line. This indicated that the nomogram exhibited good calibration in the training and validation sets, demonstrating strong consistency between the predicted and actual risks.

### 3.6 Evaluation of the clinical practicality of the risk prediction

**Figure 4:**
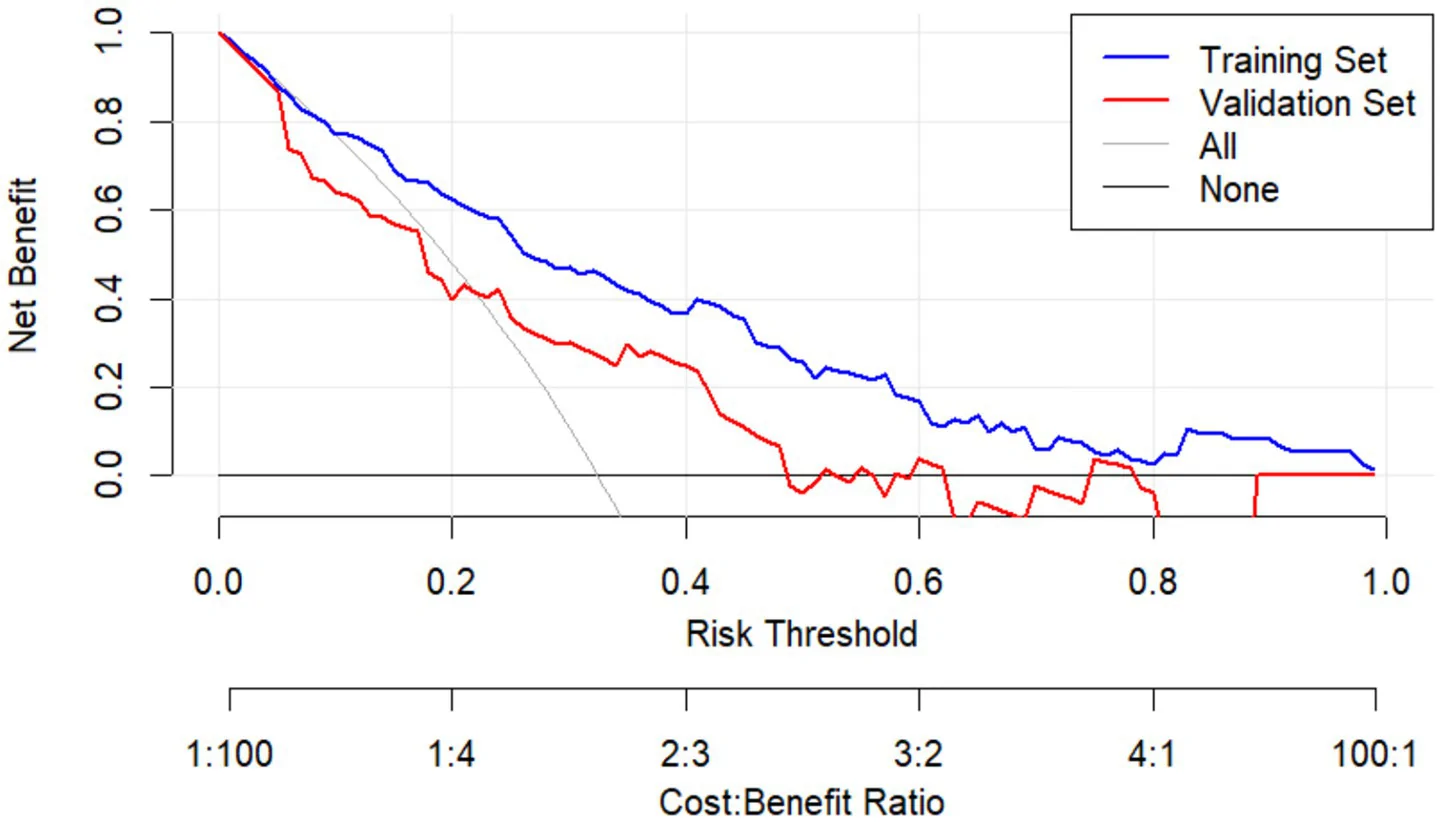
Decision curve analysis of the LASSO regression risk prediction model for WRMSDs LASSO, least absolute shrinkage and selection operator; WRMSDs, work-related musculoskeletal disorders.

The DCA showed that in the training set, when the risk threshold ranged from 2 to 10%, the net benefit of the nomogram gradually decreased from 0.95 to 0.00; however, the net benefit remained consistently higher than the two reference strategies of “treat-all” and “treat-none.” In the validation set, net benefit outperformed the reference lines across the same threshold range (Figure 4). These findings indicated that the nomogram provided a clear clinical net benefit over a wide range of risk thresholds, demonstrating good clinical applicability.

## 4 Discussion

Our findings showed that the combined prevalence of cervical, shoulder, and lumbar WRMSDs among construction workers at a hydropower station on the Qinghai-Tibet Plateau was 31.1% (see Supplementary Figure 1). In comparison, Weiner Santos et al. (19) reported a pooled overall prevalence of WRMSDs of 59% across all body sites among construction workers in their systematic review, which was as high as 63% in Asian populations. This discrepancy may be related to the occupational composition characteristics of the sample, in which civil engineering workers (with higher prevalence rates (>50%) (19)) accounted for 26.5% (86/325), whereas administrative management personnel constituted a high proportion (16.0%). Notably, administrative management personnel were mostly engaged in static work, with a prevalence rate as high as 53.8%. This unexpected finding suggests that in plateau environments, the risk of musculoskeletal disorders in non-manual labor positions should not be overlooked, and is potentially attributable to factors such as prolonged sitting, poor posture, and high psychological load that may exacerbate musculoskeletal injuries (20, 21).

We identified five independent predictive factors for WRMSDs, including sleep duration, workplace temperature, history of chronic disease, type of work, and household registration type; a corresponding risk prediction model was fitted. In-depth analysis revealed that the influence of demographic characteristics on WRMSDs exhibits particularities. Urban household registration was identified as an independent risk factor for WRMSDs (*OR* = 2.24), which differed from the conclusion of a higher risk associated with rural household registration in plain areas (22). This may be because rural registered workers are mostly local high-altitude inhabitants or long-term residents, whereas urban registered workers are primarily migrants from the plains. Studies have shown that compared with native highlanders, low-altitude migrants are more prone to neuromuscular fatigue and have a higher risk of musculoskeletal injuries. Hypoxia aggravates oxidative stress and inhibits tissue repair (23, 24).

Regarding health and environmental factors, history of chronic disease was the most strongly associated with WRMSDs in this study (*OR* = 6.02). The number of participants with chronic diseases was small, resulting in a wide CI for this variable. However, model verification confirmed that the effect was stable and significant. Studies have shown that a plateau environment further impairs the physical regulatory capacity of patients with chronic diseases and increases the compensatory load on the musculoskeletal system. Meanwhile, the side effects of long-term medication may disrupt normal muscle function; therefore, this population faces a markedly higher risk of musculoskeletal injuries at the same labor intensity (24, 25). Workplace temperature significantly influenced WRMSDs risk: compared to a comfortable environment, workers feeling cold had a significantly increased risk (*OR* = 2.47), consistent with the findings of Farbu et al. (26). This suggests that enhancing thermal insulation measures is crucial in plateau construction. An epidemiological survey indicated that people in the colder northern regions of China have a higher prevalence of knee and lower back muscle pain. A study on WMSDs among miners showed that cold working environments and damp work uniforms are more likely to cause lower back pain in workers (27, 28). Additionally, adequate sleep served as a protective factor; its physiological repair mechanisms are important for alleviating muscle fatigue and reducing injury risk (29).

Construction workers in this hydropower project have diverse types of work, which, as an important predictive factor, exerts an independent effect on the risk of WRMSDs under the special plateau working mode. Electromechanical installation workers had a significantly higher risk, which may be associated with frequent repetitive work, prolonged awkward postures, and vibration exposure. Tasks such as welding, wiring, and equipment assembly often involve sustained muscle tension and excessive limb loading, which leads to muscle fatigue and chronic strain. Sharma et al. conducted cross-sectional surveys and biomechanical modeling among mining workers and found that long-term whole-body vibration exposure and repetitive work can significantly elevate the risk of musculoskeletal injuries and induce spinal overload and tissue degeneration (12, 13). Other job categories were also identified as significant risk factors. Workers in these posts usually undertake diverse casual work with unstandardized operations. In combination with a harsh plateau environment, the musculoskeletal system is more vulnerable to cumulative damage (30).

This study has some limitations. First, the cross-sectional survey design makes it difficult to establish causal associations, as it only reveals associations at a specific point in time. Secondly, the study may be subject to the “healthy worker effect,” which potentially leads to risk underestimation. Third, the survey was conducted during a severely cold period on the plateau, which may have introduced seasonal bias. Finally, the model only underwent internal validation. Future longitudinal studies should further confirm these causal relationships and validate the model’s generalizability across populations in different regions.

In conclusion, our findings offer practical tools for early risk identification of WRMSDs among hydropower station workers on the Qinghai-Tibet Plateau, and provide scientific basis for improving occupational health management in large-scale engineering construction projects in plateau regions.

Based on the above findings, the following measures are recommended for the prevention and control of WRMSDs among plateau construction populations: First, differentiated occupational protection focusing on ergonomic improvements and psychological counseling for administrative personnel should be implemented; Second, workplace environmental management should be optimized, particularly strengthening cold protection measures and promptly improving the microclimate at work sites; Third, health management and screening should be enhanced, plateau adaptability assessments should be conducted for workers relocating from low-altitude regions, monitoring of individuals with chronic diseases should be prioritized, and health education should be carried out regularly; Fourth, work organization systems should be improved, working hours and labor intensity should be appropriately regulated, shift mechanisms should be optimized, and adequate sleep should be prioritized for workers to reduce occupational injury risks.

## Data Availability

The raw data supporting the conclusions of this article will be made available by the authors, without undue reservation.
