# Correction: Construction and evaluation of a risk prediction model for work-related musculoskeletal disorders among construction workers at a hydropower station on the Qinghai-Tibet Plateau: a cross-sectional study

**DOI:** 10.3389/fpubh.2026.2008004

**Published:** 2026-10-06

**Authors:** Anran Jiang, Yaoqi Li, Deji Zhaxi, Duxuan Zhong, Yanbiao Bai, Xingzhang Luo, Ci Song

**Affiliations:** Medical College, Xizang University, Lhasa, China

**Keywords:** construction workers, LASSO regression, nomogram, Qinghai-Tibet Plateau, work-related musculoskeletal disorders (WRMSDs)

An incorrect grant number was provided for the Department of Science and Technology of the Xizang Autonomous Region. The correct number is zdbs202215.

The original version of this article has been updated.

