# Supplementary material for: Construction and evaluation of a risk prediction model for work-related musculoskeletal disorders among construction workers at a hydropower station on the Qinghai-Tibet Plateau: a cross-sectional study
Source: Front Public Health. 2026 Jul 2;14:1850053. doi: 10.3389/fpubh.2026.1850053 (PMC13373078; doi:10.3389/fpubh.2026.1850053)
Supplement: Supplementary file 1 [file Table_1.DOCX]

Supplementary Material

# Supplementary Figures and Tables

## Supplementary Tables

Supplementary Table 1 Comparison of baseline characteristics between the training set and validation set

| **Variables** | **Training set（n=227）** | **Validation set(n=98)** | **Chi-square (χ^2^)/Z** | ***P*-value** |
| --- | --- | --- | --- | --- |
| Sex |  |  | χ^2^=0.044 | 0.834 |
| Male | 210(92.5) | 90(91.8) |  |  |
| Female | 17(7.5) | 8(8.2) |  |  |
| Age | 41(33,50) | 38(32,49) | Z=-1.162 | 0.245 |
| Ethnicity |  |  | χ^2^=1.097 | 0.578 |
| Han | 196(86.3) | 88(89.8) |  |  |
| Yi | 15(6.6) | 6(6.1) |  |  |
| Other | 16(7.0) | 4(4.1) |  |  |
| Registration place Type |  |  | χ^2^=0 | 0.995 |
| Rural | 190(83.7) | 82(83.7) |  |  |
| Urban | 37(16.3) | 16(16.3) |  |  |
| Education Level |  |  | χ^2^=2.841 | 0.417 |
| Primary school and lower | 39(17.1) | 20(20.4) |  |  |
| Junior high school | 98(43.2) | 33(33.7) |  |  |
| High school/Secondary school | 32(14.1) | 18(18.3) |  |  |
| Junior college and higher | 58(25.6) | 27(27.6) |  |  |
| Marital Status |  |  | χ^2^=3.632 | 0.163 |
| Unmarried | 41(18.1) | 24(24.5) |  |  |
| Married | 169(74.4) | 71(72.4) |  |  |
| Other | 17(7.5) | 3(3.1) |  |  |
| Personal Monthly Income |  |  | χ^2^=3.253 | 0.197 |
| ≤7000 | 56(24.7) | 30(30.6) |  |  |
| 7001–10000 | 115(50.7) | 39(39.8) |  |  |
| ≥10001 | 56(24.6) | 29(29.6) |  |  |
| Physical Exercise |  |  | χ^2^=7.468 | 0.113 |
| Never | 93(41.0) | 48(49.0) |  |  |
| 1–3 times per quarter | 31(13.7) | 18(18.4) |  |  |
| 2–3 times per month | 33(14.5) | 15(15.3) |  |  |
| 1–2 times per week | 40(17.6) | 12(12.2) |  |  |
| ≥3 times per week | 30(13.2) | 5(5.1) |  |  |
| Smoking Status |  |  | χ^2^=2.227 | 0.328 |
| Non-smoker | 102(44.9) | 40(40.8) |  |  |
| Smoker | 111(48.9) | 55(56.1) |  |  |
| Quitted smoking | 14(6.2) | 3(3.1) |  |  |
| Drinking status |  |  | χ^2^=3.502 | 0.321 |
| Never drink | 86(37.9) | 42(42.9) |  |  |
| ≤1 time per month | 56(24.7) | 29(29.6) |  |  |
| 2–4 times per month | 63(27.8) | 18(18.4) |  |  |
| ≥2 times per week | 22(9.7) | 9(9.2) |  |  |
| Type of work |  |  | χ^2^=2.442 | 0.785 |
| Civil | 63(27.8) | 23(23.5) |  |  |
| Electromechanical | 38(16.7) | 13(13.3) |  |  |
| Machine Ops | 14(6.2) | 6(6.1) |  |  |
| General Support | 43(18.9) | 22(22.4) |  |  |
| Admin | 33(14.5) | 19(19.4) |  |  |
| Other | 36(15.9) | 15(15.3) |  |  |
| High-altitude work duration (months) | 6(3,12) | 7(3,13) | Z=-0.319 | 0.749 |
| History of chronic diseases |  |  | χ2=0.322 | 0.761 |
| None | 217(95.6) | 95(96.9) |  |  |
| Yes | 10(4.4) | 3(3.1) |  |  |
| Sleep duration (h) | 7.5(6,8) | 7(7,8) | Z=-0.401 | 0.688 |
| Weekly working hours (h) | 63(56,84) | 63(55,77) | Z=-0.635 | 0.526 |
| Workplace temperature |  |  | χ2=2.405 | 0.300 |
| Comfortable | 116(51.1) | 45(45.9) |  |  |
| Hot | 49(21.6) | 29(29.6) |  |  |
| Cold | 62(27.3) | 24(24.5) |  |  |
| Work environment noise |  |  | χ2=0.001 | 0.982 |
| No impact | 199(87.7) | 86(87.8) |  |  |
| Impairs work | 28(12.3) | 12(12.2) |  |  |
| average heart rate | 82(73,90) | 82(73,91) | Z=-0.153 | 0.878 |
| average blood oxygen | 92(91,94) | 92(91,94) | Z=-1.073 | 0.283 |
| average systolic blood pressure | 130(119,142) | 129(121,143) | Z=-0.253 | 0.800 |
| average diastolic blood pressure | 84(76,94) | 84(76,95) | Z=-0.294 | 0.769 |
| bone mineral density (BMD) |  |  | χ^2^=0.037 | 0.981 |
| normal bone mass | 116(51.1) | 49(50.0) |  |  |
| osteoporosis | 14(6.2) | 6(6.1) |  |  |
| osteopenia | 97(42.7) | 43(43.9) |  |  |
| binary classification of ECG |  |  | χ^2^=0.142 | 0.706 |
| normal ECG | 169(74.4) | 71(72.4) |  |  |
| abnormal ECG | 58(25.6) | 27(27.6) |  |  |

## Supplementary Figures

##
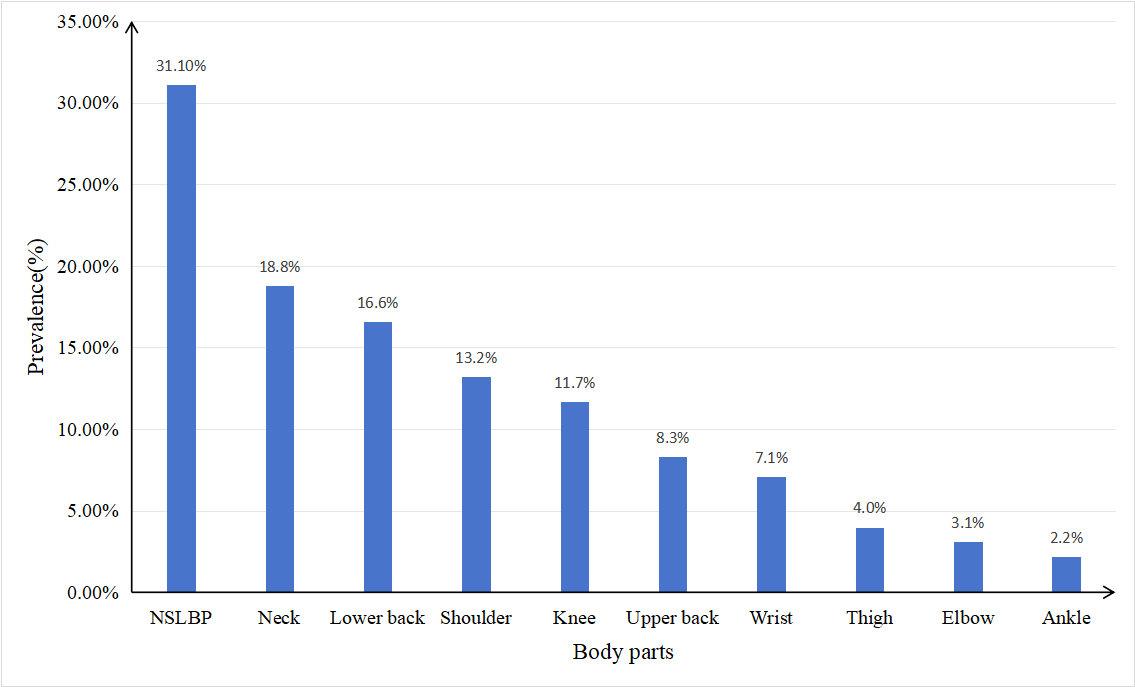
**Supplementary Figure 1.** Prevalence of work-related musculoskeletal disorders (WRMSDs) across different body sites among construction workers at a hydropower station on the Qinghai-Tibet Plateau (n=325). NSLBP: Neck, shoulder, and lower back combined prevalence
